# Global hotspots and trends in gut mycological research: a visual analytics and bibliometric approach

**DOI:** 10.3389/fimmu.2024.1457913

**Published:** 2024-10-02

**Authors:** Wenhao Zhu, Jiayu Chi, Yongde Zhang, Dongliang Wu, Xinyu Xia, Xingyu Liao, Kexin Xu, Wenying Shi, Haowen Hu, Wei Wang, Zhiyuan Lu, Zixu Zhang, Yang Liu

**Affiliations:** ^1^ Medical School of Southeast University, Nanjing, China; ^2^ Department of Gastroenterology and Endoscopy Center, Xining Hospital of Traditional Chinese Medicine, Xining, China; ^3^ Department of Gastroenterology and Endoscopy Center, Binhai County Second People’s Hospital, Yanchen, China; ^4^ College of Textile and Clothing Engineering, Soochow University, Suzhou, Jiangsu, China; ^5^ Beijing Jishuitan Hospital, Beijing, China; ^6^ Department of Gastroenterology and Endoscopy Center, Pukou People’s Hospital, Nanjing, China; ^7^ Department of Gastroenterology, Zhongda Hospital, Southeast University, Nanjing, China

**Keywords:** gut, microbiota, gut microbiome, fungi, gut fungal ecology

## Abstract

**Background:**

Recent findings highlight the significant impact of intestinal fungi on the complex makeup of the gut microbiota and human health, challenging past oversights. However, a lack of thorough systematic and quantitative analyses remains. This study aims to address this gap by thoroughly examining the current research on gut fungi. Through analyzing developments and unique features in this area, our goal is to foster a deeper understanding and identify future research pathways.

**Methods:**

We performed an extensive bibliometric analysis on documents from 2000 to 2023, sourced from the Web of Science Core Collection (WoSCC). Utilizing advanced visualization tools such as VOSviewer, CiteSpace, and Bibliometrix R, we meticulously examined and illustrated the data in scientific landscapes and networks.

**Results:**

A total of 1434 papers were analyzed, revealing a substantial increase in publication volume over the past two decades, particularly in 2020. Contributions came from 67 countries, 2178 institutions, and 8,479 authors. China led in publication output with 468 articles, followed by the University of California with 84 articles, and ZHANG F as the most prolific author with 17 articles. Emerging research areas such as “Fungal-Bacteria Interactions,” “Gut Fungus and Gut-Brain Axis,” and “Gut Fungus and Immunity” are expected to attract growing interest in the future.

**Conclusion:**

This extensive bibliometric analysis offers a current overview of scholarly efforts concerning intestinal fungi, highlighting the predominant landscape in this field. These insights can assist scholars in identifying appropriate publication avenues, forming collaborative relationships, and enhancing understanding of key themes and emerging areas, thereby stimulating future research endeavors.

## 1 Introduction

The gastrointestinal tract harbors a diverse array of microorganisms, collectively referred to as the gut microbiota, which includes bacteria, viruses, fungi, and archaea. The intricate gut microbiota plays a pivotal role in human health and disease, primarily exerting its influence in areas such as host digestion, the normal metabolism of various substances, the synthesis of essential vitamins, the recognition and resistance to pathogens, the maintenance of functional stability of the intestinal barrier, and the shaping and regulation of the immune system (1–3).

In previous studies, the primary focus of research on the gut microbiome has predominantly been on bacterial components. This is likely due to the relatively low abundance of fungi within the gut microbiome, which typically ranges from 0.1% to 1% (4). However, in recent years, scientists have progressively unveiled the non-negligible role of fungi, highlighting their significance in the complex ecosystem of the gut (5).Certain pathogenic fungal species have been implicated in various disorders, including cancer, inflammatory bowel disease, immune dysregulation, and atherosclerosis (6). Concurrently, commensal fungi may leverage pattern recognition receptors such as Dectin-1 and TLRs to exert significant roles in crucial physiological pathways, including the gut-brain axis, metabolic equilibrium, and immune modulation. For example, in 2019, the groundbreaking findings by Bacher and colleagues have elucidated that, within a cohort of 30 fungal community members, Candida albicans stands out as the most efficacious agonist for the induction of human CD4+ memory T helper 17 (Th17) cells (7). In parallel, the field of gut mycobiome research still harbors significant gaps that warrant exploration. These include the definitional issues surrounding the gut mycobiome, the taxonomic ascertainment of intestinal fungal species, and their contributions to human health and the pathogenesis of diseases (8).

In response to the vast amount of literature data in the field of gut mycobiome, we have considered bibliometric analysis, an emerging research methodology. Bibliometric analysis, a quantitative method for evaluating the impact and trends of academic publications, involves statistical examination of citations, author collaborations, and journal metrics (9). Traditional reviews, while offering a comprehensive overview of a particular field, often lack specific data support and visualization, requiring readers to possess a high level of expertise. Compared to them, bibliometric analysis offers a rigorous approach to understanding vast amounts of unstructured data, which can be employed to decipher and map the accumulated scientific knowledge and evolutionary nuances of established fields. Within the realm of gut mycobiome research, it facilitates scholars in acquiring a holistic overview at once, pinpointing knowledge gaps, formulating innovative research directions, and strategically positioning their anticipated contributions to the field (10).

Historically, bibliometric analyses within this domain have predominantly encompassed the gut microbiome, without specifically highlighting the role of fungi. The present study endeavors to elucidate the significant function of fungi within the gut microbiome by leveraging data from the Web of Science, employing visualization to delineate the distribution of publications, authors, institutions, and keywords from 2000 to 2023. By identifying trends and research foci, this analysis aims to systematically articulate the study of the gut mycobiome in physiological and pathological mechanisms and its association with clinical diseases.

## 2 Methods

### 2.1 data collection

Data on Gut microbiota and fungi were retrieved from the Science Citation Index Expanded (SCI-EXPANDED) and the Social Sciences Citation Index (SSCI) between January 1, 2000, and December 31, 2023. These datasets were extracted from the Web of Science Core Collection (WoSCC) database on February 20, 2024, using advanced search terms. The construction of advanced search terms involves the strategic combination of Boolean operators, synonyms, wildcards, and filters to precisely and efficiently retrieve relevant literature or data within a specific domain.

1. ((((((TS=(gut microbiota)) OR TS=(intestinal microbiota)) OR TS=(fecal microbiota)) OR TS=(gastrointestinal microbiota)) OR TS=(gut microbiome)) OR TS=(intestinal microbiome)) OR TS=(fecal microbiome)
2. ((TS=(fungus)) OR TS=(fungoid)) OR TS=(fungi)
3. #2 AND #1

All data elements, encompassing titles, keywords, authorship, geographical and institutional origins, publishing journals, publication dates, H-indices, and citation metrics, were meticulously extracted from the publications identified by the two authors (ZW and CJ). The search formula we applied yielded an initial corpus of 6,267 documents pertinent to our research theme. After imposing restrictions on document types, language preference for English, and temporal constraints, the collection was refined to 2,109 documents.

The subsequent phase involved a meticulous manual curation by the same authors to exclude articles that did not align with the thematic focus of our study. Discrepancies in the assessment were amicably addressed by an esteemed corresponding author (LY), following a systematic procedure. This procedure commenced with a critical analysis of the titles and abstracts to ascertain the thematic relevance of the articles. Those deemed suitable based on this initial evaluation were subjected to a thorough manual review to confirm their alignment with our research objectives.

**Figure 1:**
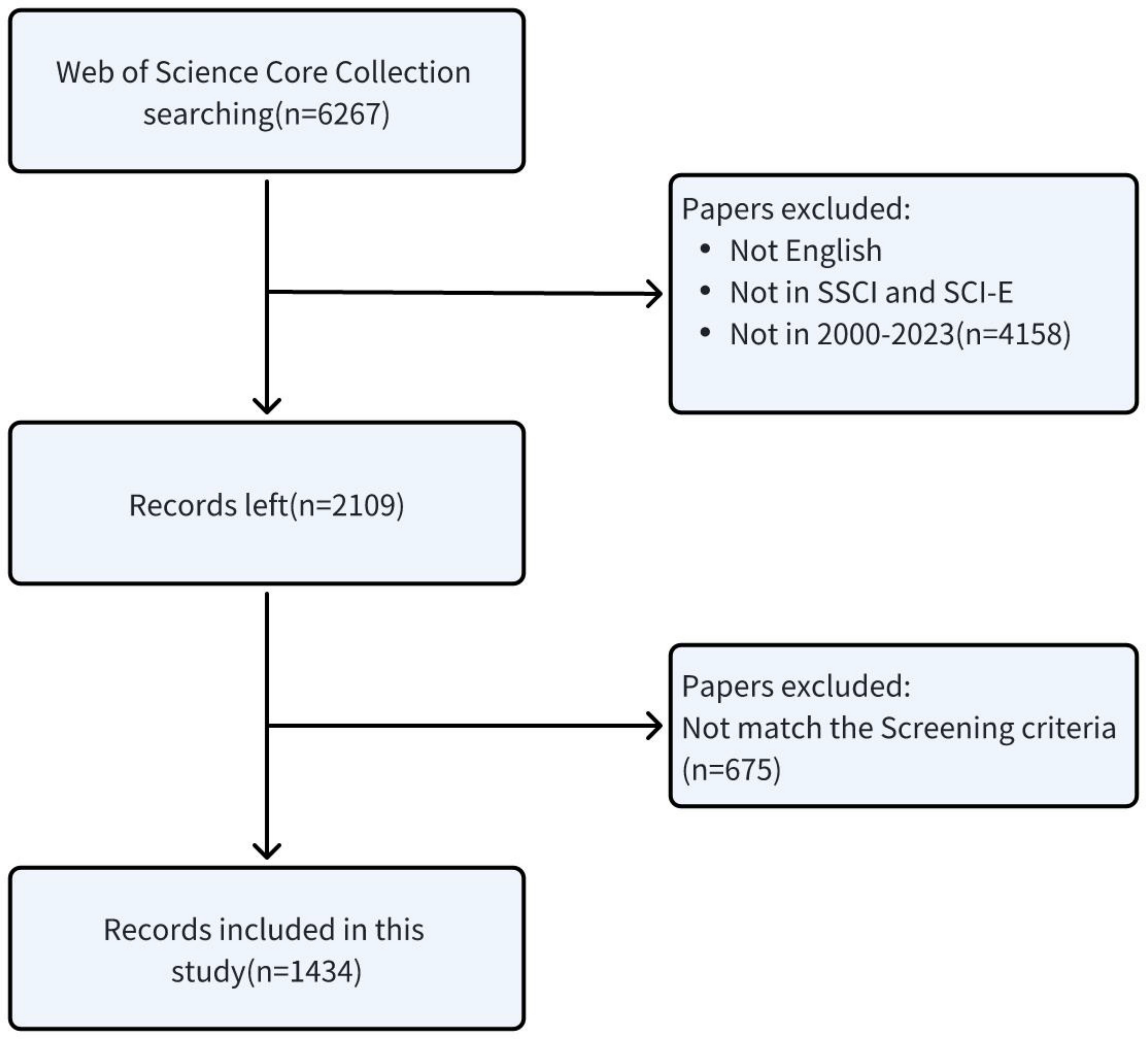
Flow diagram of literature search and screening.

In instances where the thematic relevance could not be ascertained from the title alone, the full texts were procured from the Southeast University Library for an in-depth examination to verify their pertinence to our study. Post the stringent application of our inclusion and exclusion criteria, a curated subset of 1,434 articles was selected for an in-depth analysis, thereby ensuring the scholarly rigor and thematic integrity of our literature review. The detailed procedures for subject enrollment and exclusion criteria are delineated in **Figure 1**.

### 2.2 Data analysis

The initial dataset was sourced from the WoSCC database and analyzed using VOSviewer (version 1.6.20), CiteSpace (version 6.1.6), and the Bibliometrix R package. These tools were selected for their specific contributions to our bibliometric analysis. VOSviewer was utilized for its network visualization capabilities, which facilitated the exploration of citation and co-citation networks. CiteSpace was employed to perform advanced co-citation analysis and to uncover trends in publication dynamics. The Bibliometrix R package provided a robust framework for data extraction and refinement within the R environment, allowing for detailed bibliographic analysis. Utilizing these three tools allows for the straightforward creation of visual representations of bibliometric data, a method widely employed in mainstream bibliometric analysis.

## 3 Results

### 3.1 Annual development trend of publications and citation

**Figure 2:**
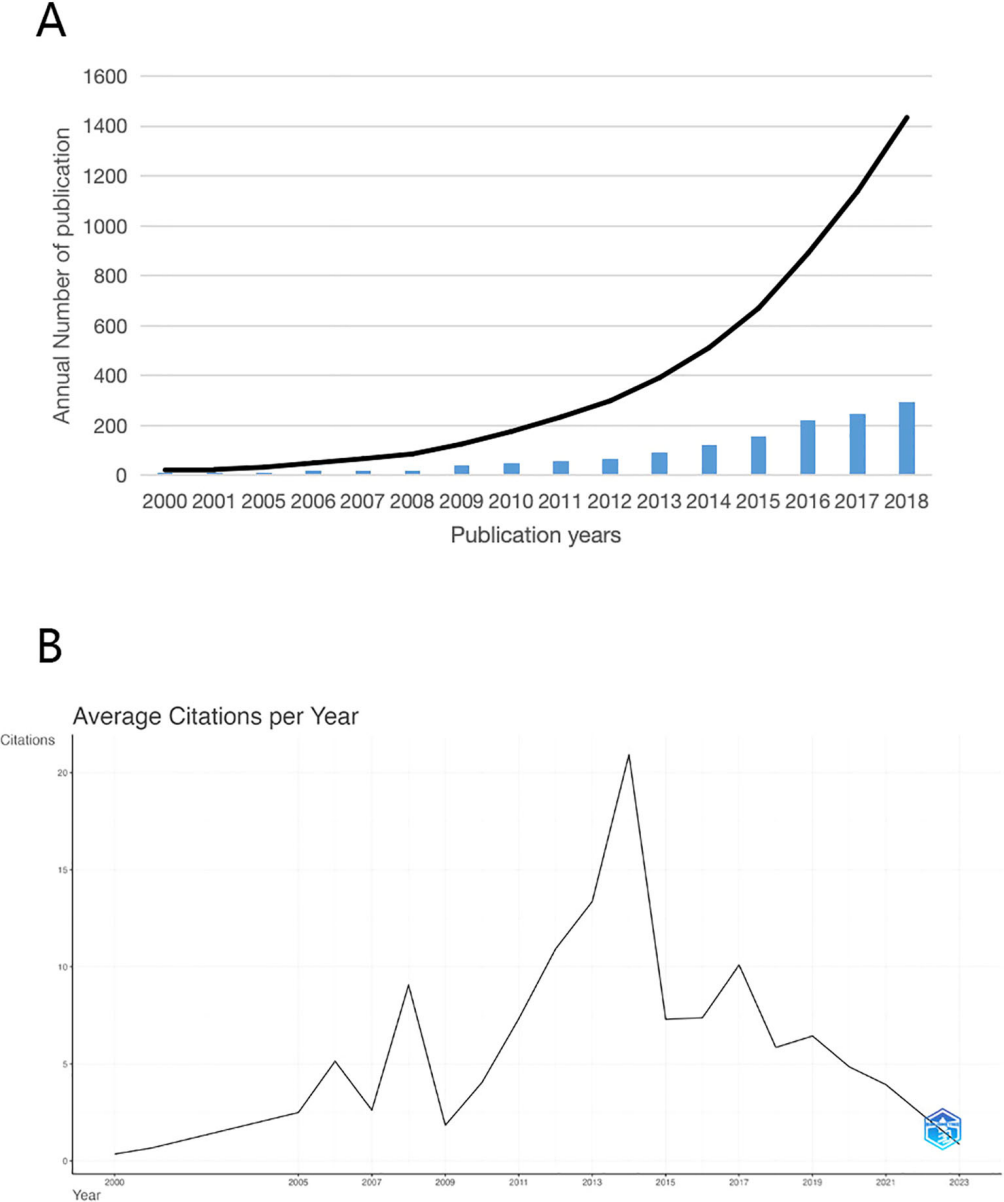
**(A)** Trends in the growth of publications in gut fungi **(B)** The number of average citations per year in gut fungi.

In **Figure 2A**, we present the annual publication volume and cumulative number of published documents spanning the past 23 years, while **Figure 2B** delineates the annual average citations within this domain. Noteworthy is the substantial increase witnessed in both annual and cumulative publications over the years. Annual citations reached their zenith in 2006, 2008, 2014, and 2017, signifying notable strides in research during these periods. Critically speaking, this could be attributed to a significant increase in the number of new journals, as well as a tendency among researchers to pursue trending fields in academia. Particularly striking is the citation peak in 2014, which may be attributed to the first clinical application of Next-Generation Sequencing-based metagenomic detection (mNGS) that successfully saved the life of a 14-year-old boy (11, 12). These trends underscore the burgeoning significance of mycology as a global focal point within the sphere of human health.

### 3.2 Analysis of productive journals

A comprehensive analysis reveals a total of 1434 articles related to gut fungi, dispersed across 506 journals. Notably, **Table 1** showcases the top ten most prolific journals in this domain, offering a glimpse into the scholarly landscape. Topping the list is Frontiers in Microbiology, boasting 104 articles, which translates to 7.25% of the total publications. Following closely behind is PLOS ONE, with 51 articles, representing 3.56% of the corpus. However, it is intriguing to note that MICROBIOME, despite ranking fourth in terms of the number of articles, stands out with an impressive impact factor (IF) of 15.5 and an average number of citations per article at 72, underscoring its substantial influence within the realm of intestinal mycology. Regarding these top-tier journals, they have a high volume of publications, and many scholars in the field will opt for these journals when submitting their work. The editorial policies of these journals can also indirectly influence the direction of research. This data not only highlights the breadth of research interest in gut fungi but also emphasizes the importance of key journals in shaping the discourse and impact of this field.

**Table 1:** The top ten journals based on total citations in gut fungal microbiota.

Sources	Articles	Citations	Citations per-publication	Journal IF
FRONTIERS IN MICROBIOLOGY	104	3001	29	5.2
PLOS ONE	51	2163	42	3.7
SCIENTIFIC REPORTS	41	1971	48	4.6
MICROBIOME	27	1939	72	15.5
MICROBIAL ECOLOGY	25	1711	68	3.6
JOURNAL OF FUNGI	23	1652	72	4.7
MICROBIOLOGY SPECTRUM	23	1428	62	3.7
MICROORGANISMS	20	1050	53	4.5
ANIMALS	18	1015	56	3
APPLIED AND ENVIRONMENTAL MICROBIOLOGY	17	973	57	4.4

### 3.3 Global meta-analysis: countries, institutions, and authors

**Figure 3:**
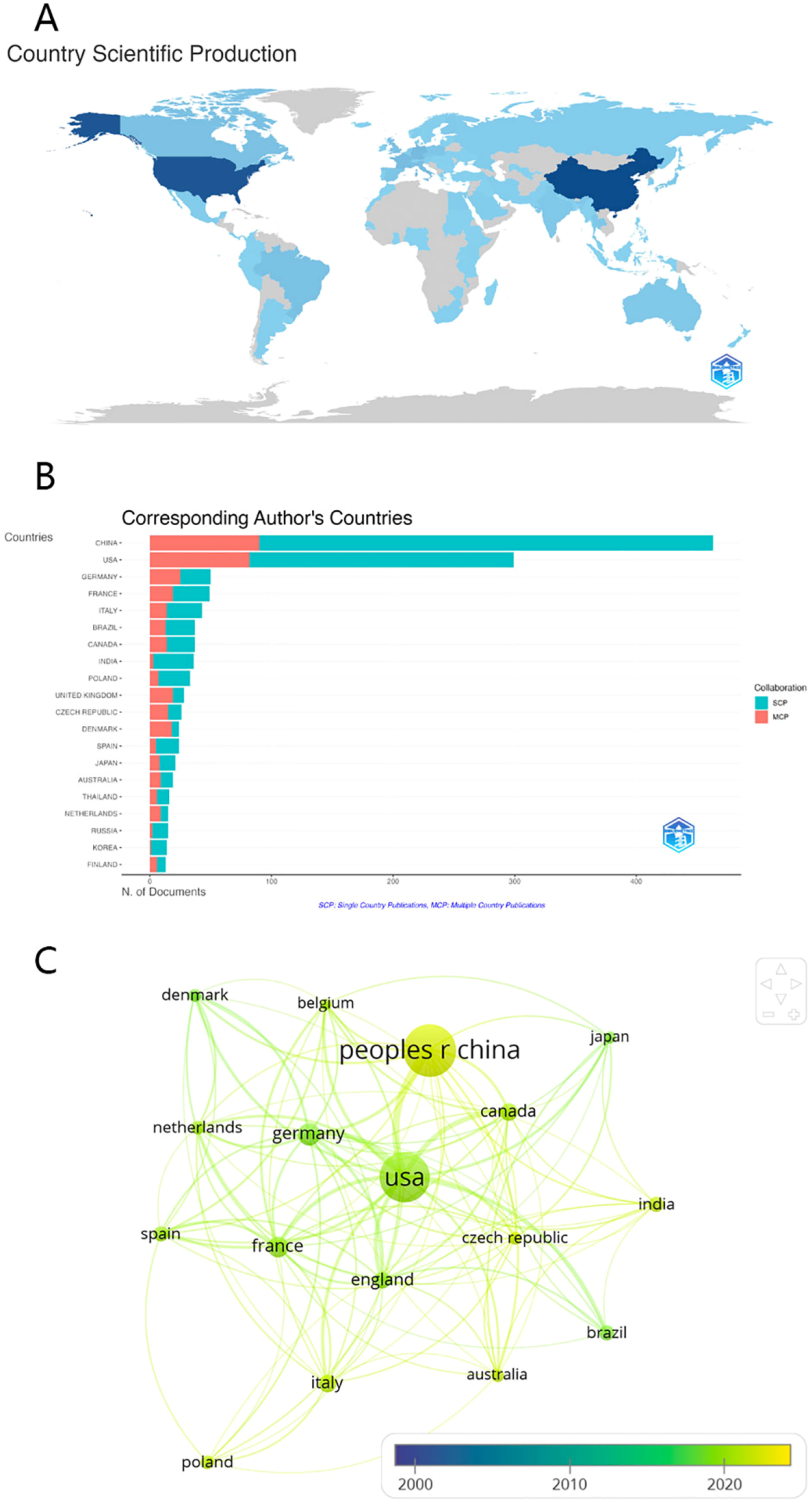
**(A)** Distribution of publications from different countries/regions **(B)** The top 20 countries with the most corresponding authors **(C)** International collaboration network of the top 20(3 excluded because of no links) countries over time.

In the expansive field of gut microbiota research, exploration into fungal elements has attracted interest from 67 countries and regions worldwide. **Table 2** delineates the publication output, highlighting that China tops the list with a total of 463 scholarly contributions, followed by the USA with a count of 299 articles. These two nations surpass the 100-article mark, indicating significant investment in fungal gut research. While China leads in volume, the USA achieves nearly five times the average citations per article, reflecting higher research impact. The leading position of China and the United States in terms of publications is primarily attributed to ample research funding and robust scientific infrastructure. Collaboration networks, illustrated in **Figure 3**, reveal the USA as the central hub for gut fungi research, closely collaborating with China, Germany, the United Kingdom, and Canada. The formation of these collaboration networks facilitates the ease with which scholars from around the globe can exchange their research directions. Consequently, whenever a new hot topic is identified, all scholars can quickly detect it and delve into further exploration. Recent years have seen a surge in international academic exchanges, demonstrating China’s substantial potential in this field.

**Table 2:** The top 10 productive countries/regions involved in gut fungi.

Country	Articles	SCP	MCP	Freq	MCP_Ratio	TC	Average Article Citations
CHINA	463	373	90	0.323	0.194	6918	14.80
USA	299	217	82	0.209	0.274	22341	74.50
GERMANY	50	25	25	0.035	0.5	1884	36.90
FRANCE	49	30	19	0.034	0.388	3408	69.60
ITALY	43	29	14	0.03	0.326	2811	65.40
BRAZIL	37	24	13	0.026	0.351	627	16.90
CANADA	37	23	14	0.026	0.378	855	23.10
INDIA	36	33	3	0.025	0.083	707	19.10
POLAND	33	26	7	0.023	0.212	365	10.70
UNITED KINGDOM	28	9	19	0.02	0.679	1085	38.80

MCP represents the number of co-authored papers with authors from other countries, SCP represents the number of co-authored papers with authors of the same nationality, MCP_Ratio reflects the rate of international cooperation, and TC usually refers to the total number of citations of a document. They are calculated based on the structure and patterns in the citation network and are used to quantify how much an article contributes to the citation network.

**Table 3** meticulously outlines the affiliations of authors involved in intestinal fungi research. The University of California System leads the chart in both article count and citations, reflecting its substantial research output and scholarly influence. Following closely is the esteemed Chinese Academy of Sciences, with 71 articles and 310 citations. The leading positions of these two institutions are likely due to their large scale, with numerous branches or faculties and corresponding teams of experts engaged in this field. Notably, Chulalongkorn University in Thailand emerges as a strong contender, securing the third position with 57 articles and 309 citations, surpassing several Western counterparts.

**Table 3:** The top 10 institutions of articles and citations involved in gut fungi.

Affiliation	Articles	Country
UNIVERSITY OF CALIFORNIA SYSTEM	84	USA
CHINESE ACADEMY OF SCIENCES	71	China
CHULALONGKORN UNIVERSITY	57	Thailand
CENTRE NATIONAL DE LA RECHERCHE SCIENTIFIQUE (CNRS)	54	France
WEILL CORNELL MEDICINE	52	USA
CORNELL UNIVERSITY	50	USA
HARVARD UNIVERSITY	50	USA
INRAE	50	France
INSTITUT NATIONAL DE LA SANTE ET DE LA RECHERCHE MEDICALE (INSERM)	46	France
UNIVERSITY OF WISCONSIN MADISON	43	USA

**Figure 4:**
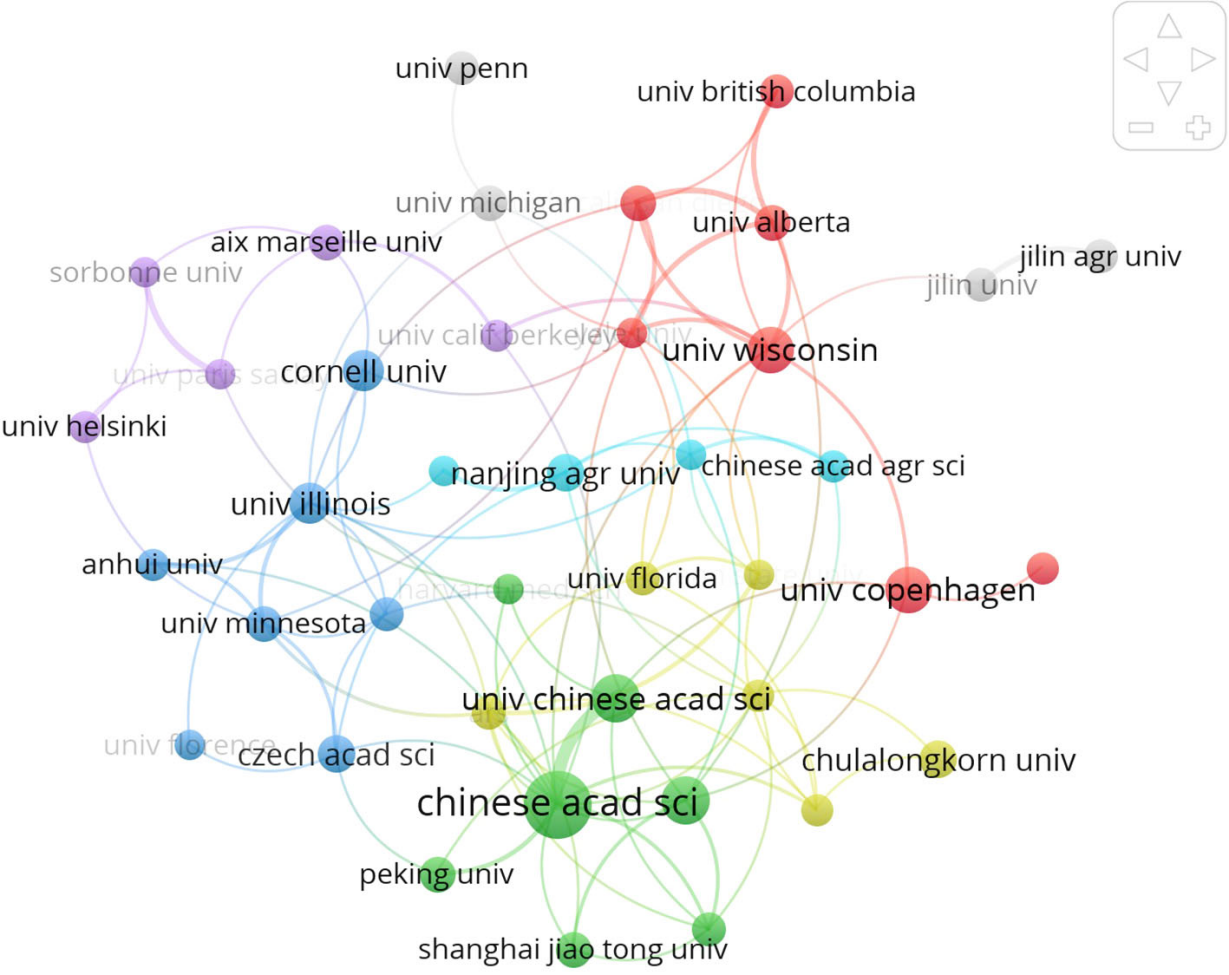
International collaboration network of the top 40 institutions in gut fungi. All institutions have been categorized into eight distinct clusters of different colors, with institutions of the same color exhibiting more profound collaboration ties.

**Figure 4** intricately illustrates the collaboration network among esteemed universities and institutions involved in intestinal fungi research, delineating 25 distinct clusters, each represented by a unique color. At the core of this collaborative ecosystem lies the Chinese Academy of Sciences, serving as the linchpin and facilitating connections with six Chinese universities and institutions. Globally, leading institutions in agriculture and medicine conduct extensive research on intestinal fungal communities, showcasing interconnectedness and emphasizing the substantial importance of intestinal fungi in both agricultural and medical spheres.

The dataset in question encompasses a formidable assembly of 8,479 authors, each a luminary within the realm of intestinal fungal research. In **Table 4**, we present a curated selection spotlighting the top 10 authors based on their H-index. It is noteworthy that Iliev Id holds the top position in both h-index and total citation rates, and commenced his investigation into fungal commensalism versus pathogenesis in 2012, thus establishing himself as a pivotal figure in the field. His seminal paper, published in *Nature Reviews Immunology* in 2014, titled “The Mycobiota: Interactions Between Commensal Fungi and the Host Immune System,” summarizes the mechanisms of interactions between symbiotic fungi, pathogenic fungi, and the immune system (13). Simultaneously, Zhang F claims preeminence in the tally of Fractionalized articles, G-index, and NP, underscoring the profound impact of his contributions to the intestinal fungal community.

**Table 4:** The academic contributions of the top ten scholars in the comprehensive evaluation score.

Element	Articles Fractionalized	h_index	g_index	m_index	TC	NP	PY_start
ILIEV ID	1.48	13	13	1	2092	13	2012
POULSEN M	1.99	11	14	1	588	14	2014
ZHANG F	2.41	11	17	1.1	665	17	2015
LEELAHAVANICHKUL A	1.75	9	12	1.125	334	12	2017
SAPOUNTZIS P	1.83	9	10	0.9	296	10	2015
BOOMSMA JJ	1.54	8	9	0.727	534	9	2014
HUBE B	0.86	8	10	1.143	626	10	2018
UNDERHILL DM	0.75	8	8	0.615	1617	8	2012
WANG Y	1.73	8	13	1.6	182	17	2020
ZHANG H	1.17	8	9	0.889	234	9	2016

Articles Fractionalized (AF) quantifies collaborative research in an author’s work. The h-index measures productivity and influence, tallying papers cited at least as often as they were published. The g-index evaluates impact through citation distribution. The m-index combines productivity and publication longevity. PY_start marks the start of an author’s scholarly contributions. These metrics, rooted in citation patterns, aid in evaluating scholarly impact and collaboration.

**Figure 5:**
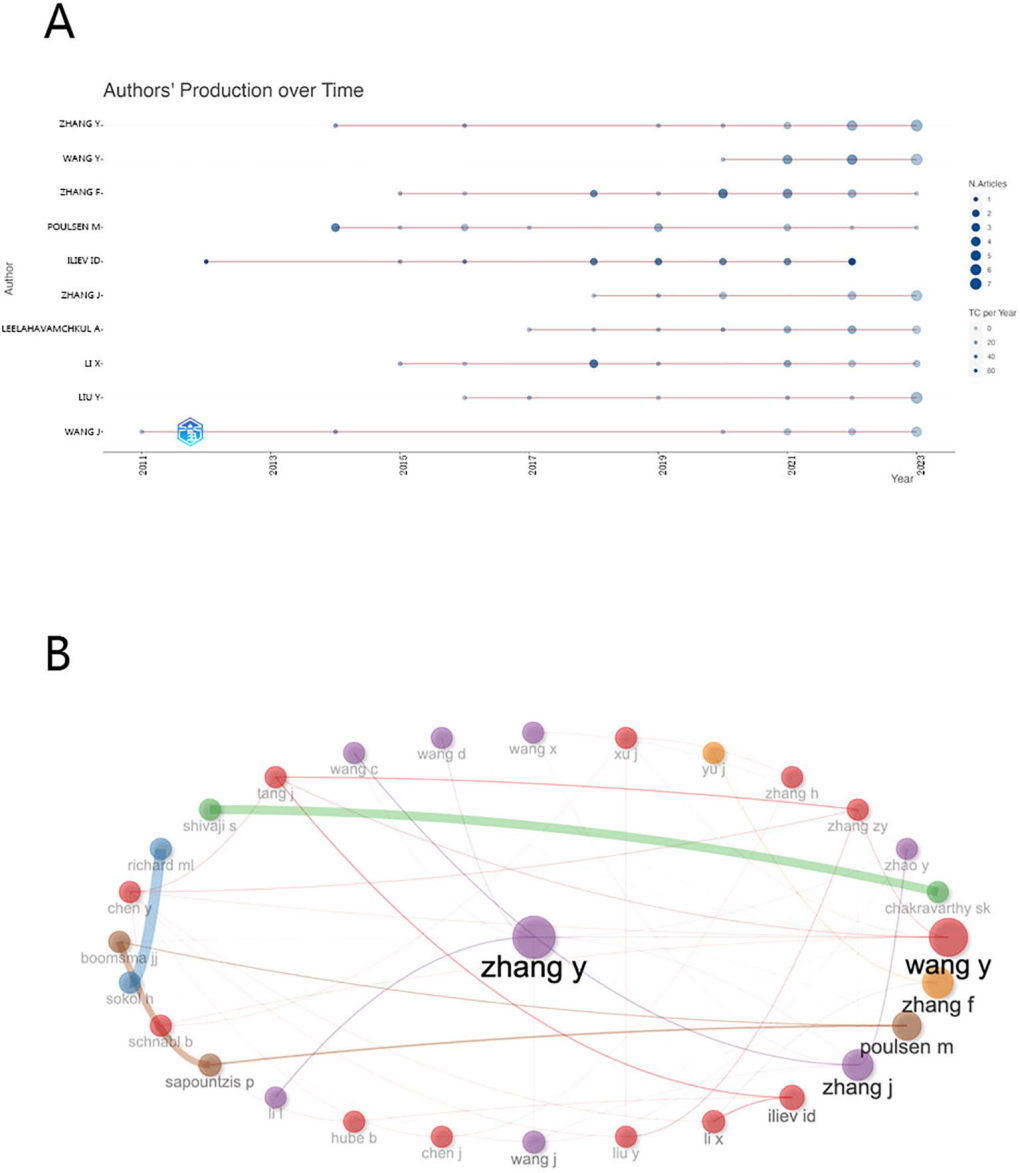
**(A)** The top 10 authors’ annual publications over time in gut fungi **(B)** The top 30 authors’ Cooperative network in gut fungal microbiome.

To illustrate the temporal evolution of scholarly productivity, our team devised a bubble chart delineating the trajectory of the top 10 most prolific authors in terms of article publication over time, as depicted in **Figure 5A**. Wang J and Iliev Id emerge as pioneers, initiating their scholarly journey during the early stages of research around 2011-2012, when discussions on the subject were limited. However, by 2014, a growing cohort of scholars had shifted their focus to gut microbiome fungi, leading to a significant increase in articles pertaining to this emerging field.

In **Figure 5B**, we provide a visual depiction of collaborative dynamics among scholars. At its core is Professor Zhang Y from Shanxi University, a distinguished expert acclaimed for his knowledge in Cordyceps sinensis (14). Intriguingly, Western scholars appear as isolated nodes with sparse connections, indicative of a dearth in collaborative research initiatives, while Chinese scholars are interconnected by a dense network of lines, symbolizing their close-knit collaborative bonds. This may be attributed to the fact that the research funding for scholars in China is predominantly sourced from the National Natural Science Foundation of China and governmental subsidies, whereas the financial backing for Western scholars is more varied, including support from government entities, private research organizations, philanthropic contributions, and sponsorships from technology corporations. As for the quality of collaborations among top authors, an excellent illustration is provided by an article on the role of fungi and immunity in inflammatory bowel disease, published in Nature in 2022 by Ellen J. Scherl and Iliyan D. Iliev (15).

### 3.4 Conditions of references and co-cited references

**Table 5** provides a comprehensive overview of the top 10 research articles, ranked by total citations from 2000 to 2023 (16–25). The leading article, authored by David L. and published in Nature, elucidates the gut microbiome’s swift adaptability to dietary changes, potentially accommodating various human dietary preferences. The 16S rRNA gene sequencing technology employed in this study, in conjunction with OTU clustering and ITS sequencing, has established itself as a fundamental method for subsequent research on gut fungal communities. This approach has also facilitated the advancement of interdisciplinary studies involving gut fungi (16). Another significant study, authored by Teresa Zelante and featured in Immunity, explores the relationship between diet and fungal and archaeal populations. It reveals the influence of dietary tryptophan on host-fungal symbiosis mediated by the microbiota (17). Subsequent entries in the studies explore various aspects of fungal involvement across different biological contexts, ranging from inflammatory bowel disease to neonatal intestinal fungal microbiota and immunity, shedding light on the intricate interplay between diet, fungi, and archaea, while also evaluating the advantages and disadvantages of culturomics and metagenomic sequencing methodologies.

**Table 5:** The top 10 most cited articles in the field of gut fungi research from 2000-2023.

Paper	DOI	Total Citations	TC per Year	Normalized TC	56Journal IF
DAVID LA, (16), NATURE	10.1038/nature12820	6047	549.73	26.25	64.80
ZELANTE T, (17), IMMUNITY	10.1016/j.immuni.2013.08.003	1422	118.50	8.86	32.40
SOKOL H, (18), GUT	10.1136/gutjnl-2015-310746	752	94.00	9.30	24.50
ILIEV ID, (19), SCIENCE	10.1126/science.1221789	750	57.69	5.28	56.90
FUJIMURA KE, (20), NAT MED	10.1038/nm.4176	676	75.11	10.18	82.90
LAGIER JC, (21), CLIN MICROBIOL INFECT	10.1111/1469-0691.12023	673	51.77	4.74	14.20
HOFFMANN C, (22), PLOS ONE	10.1371/journal.pone.0066019	513	42.75	3.20	3.70
LEWIS JD, (23), CELL HOST MICROBE	10.1016/j.chom.2015.09.008	509	50.90	6.97	30.30
SARTOR RB, (24), GASTROENTEROLOGY	10.1053/j.gastro.2016.10.012	503	62.88	6.22	29.40
NASH AK, (25), MICROBIOME	10.1186/s40168-017-0373-4	485	60.63	6.00	15.50

**Figure 6:**
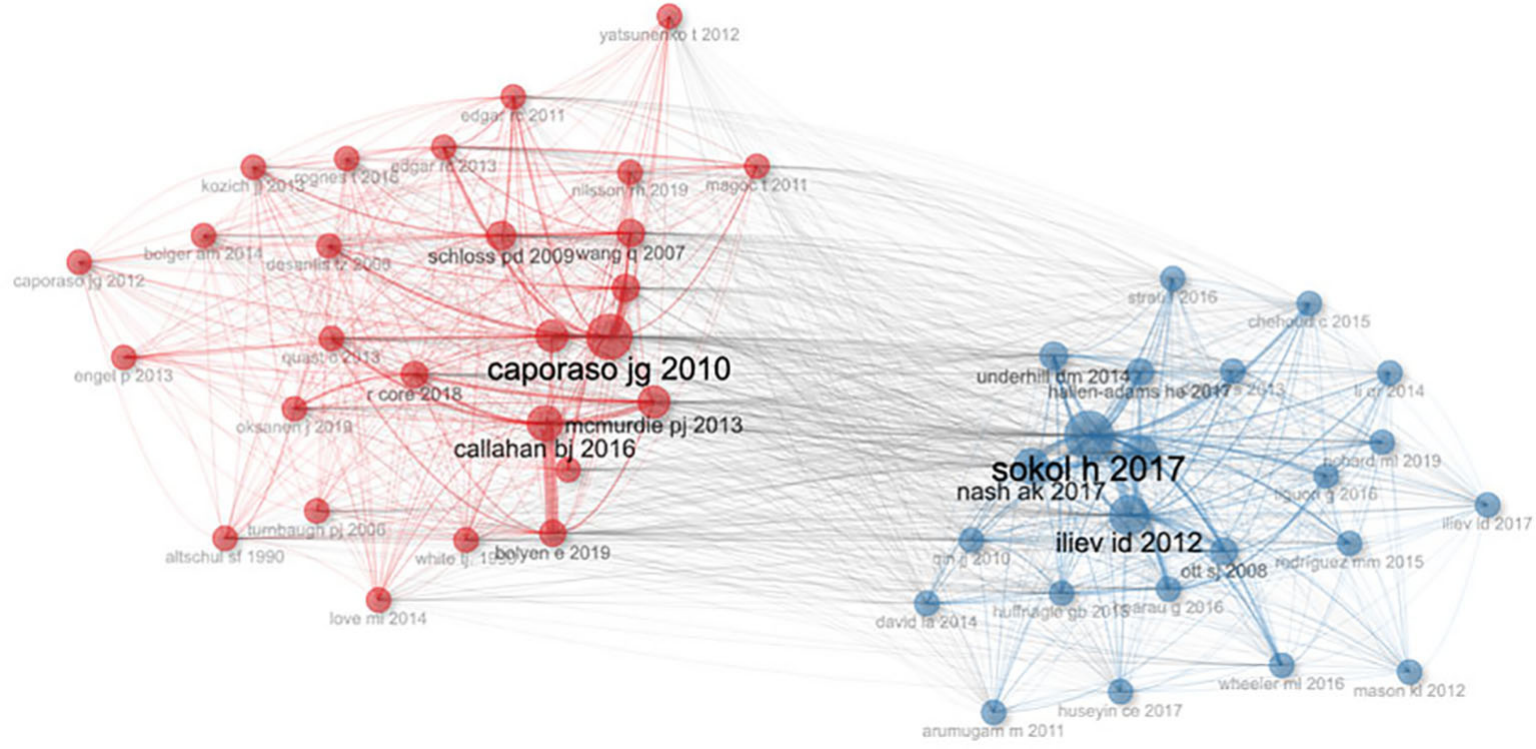
Co-citation Network of gut fungal microbiome.

Co-citation refers to the phenomenon where two or more documents are cited together by a third document, indicating their relatedness or relevance in a specific research context. The concept of “co-citation of references” emerges as a valuable tool for understanding the interconnectedness of scholarly discourse, indicating instances where multiple papers are cited together in subsequent works (26). **Figure 6** illustrates six papers with particularly strong co-citation relationships, authored by Caporaso JG 2010, Sokol H 2017, Nash AK 2017, Iliev ID 2012, Callahan BJ 2016, and McMurdie PJ 2013. These papers hold prominent positions within the top ten list, highlighting the symbiotic relationship between co-citation and scholarly influence. From the network relationships, it is also clear that research on gut fungi is divided into two schools of thought: one focusing on the connection between gut fungi and the immune system, and the other dedicated to expanding the diversity of gut fungal communities.

### 3.5 Keyword visualization and evolutionary trend analysis

**Figure 7:**
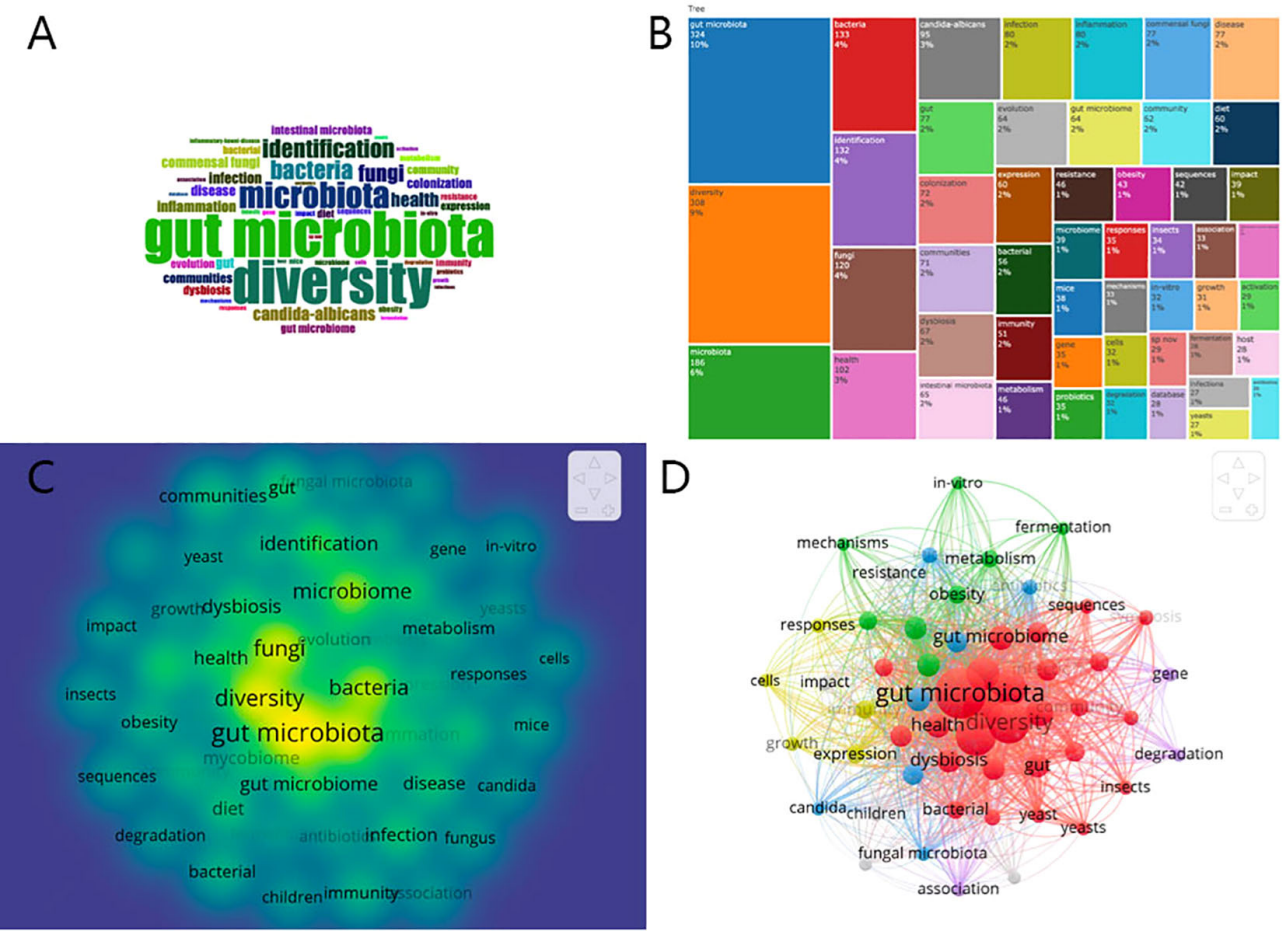
**(A)**Distribution of top 50 Keywords in the form of WordCloud. **(B)** Treemap chart showing the proportion of the top 50 keywords. **(C)** Fluorescence heat map of top 50 Keywords. **(D)** Cluster analysis of high-frequency keywords (frequency ≥15) based on all keywords of publications in gut fungal microbiota (different colors represent different clusters, the size of the circle represents the frequency the keywords appear, and the thickness of the line represents the total link strength between keywords).

In this study, keywords extracted from titles and abstracts served as proxies for authors’ primary themes and research. A total of 3782 keywords were collected from the reviewed papers. The top 50 most recurrent keywords were then visualized using word clouds and treemaps, with the word cloud presented in **Figure 7A**, the treemap in **Figure 7B**, and the heat map in **Figure 7C**, facilitated by the R tool’s Bibliometrix packages. The analysis revealed prevalent terms such as “gut microbiota,” “diversity,” “microbiota,” “bacteria,” “identification,” “fungi,” “health,” and “Candida albicans,” among others, highlighting their frequent occurrence as both search terms and research emphases. This observation carries significant implications. For instance, the concurrent appearance of keywords such as “gut microbiota” and “diversity” likely reflects the current research emphasis on enhancing the diversity of gut microbiota. This focus has, in turn, facilitated advancements in the study of gut fungi.

A clustering analysis was conducted using VOSviewer to assess the interconnections among identified keywords, leveraging both the frequency of co-occurrence in publications and the robustness of their associations. Keywords were clustered into distinct categories, each denoted by a unified color scheme. As shown in **Figure 7D**, keywords were segregated into five clusters. Cluster 1, the “red topic” with 27 items, highlights the diversity within the gut microbiota, emphasizing the interplay between gut fungi and bacteria. Cluster 2, the “green topic” with 8 items, explores the relationship between intestinal fungi and diseases such as inflammation and obesity, alongside biochemical processes like fermentation and metabolism. Cluster 3, marked by an azure hue with 7 items, focuses on fungal microbiota colonization, particularly organisms like Candida albicans. Cluster 4, colored yellow with 5 items, examines immune cell responses influenced by fungi. Cluster 5, the “purple topic” with 3 items, investigates gene degradation within gut fungi. The connections observed between nodes within these clusters highlight a significant level of keyword co-occurrence, illustrating the complex web of interactions that characterizes this research field.

**Figure 8:**
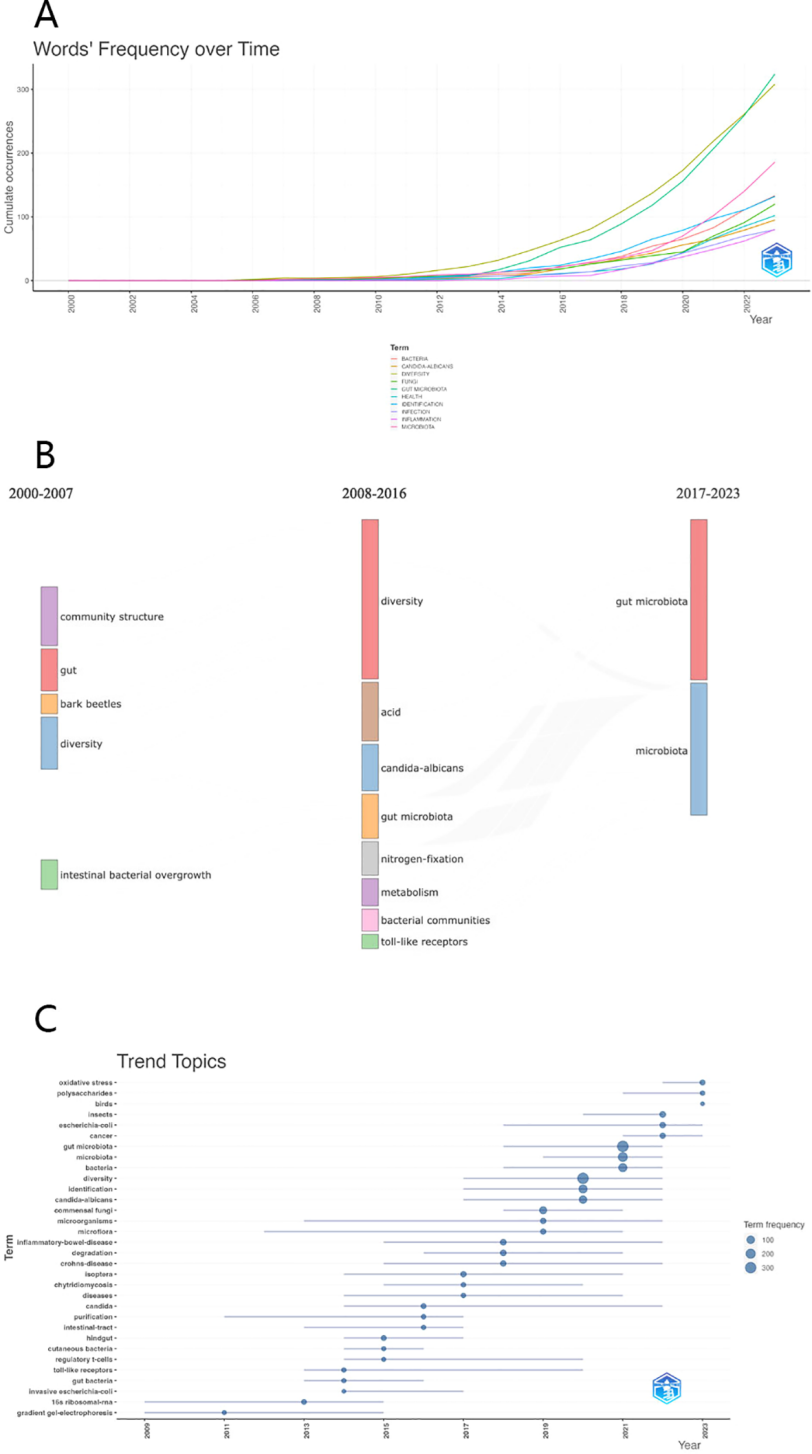
**(A)** The heat map illustrating the fluctuation in popularity of the top ten keywords from the year 2000 to 2023. **(B)** The Sankey diagram showing the key terms across three developmental phases in the field of intestinal fungi: **(C)** Bubble chart indicating the change of keywords over time and the keyword explosion.

The evolution and trajectory of keywords serve as a gauge, providing insights into the frontiers of knowledge progression. **Figure 8A** meticulously delineates the fluctuations in citation frequencies of the top ten frequently used keywords spanning from 2000 to 2023. Notably, the simultaneous surge in occurrences of “gut microbiota” and “diversity” highlights a parallel and rapid ascent, indicative of a close interconnection between them. The presence of keywords such as “health,” “bacteria,” and “infection” underscores the broader interdisciplinary integration of gut fungal microbiome research with fields such as bacteriology and clinical medicine.

Utilizing Citespace, we imported and analyzed keyword trend data, resulting in the generation of a comprehensive Sankey diagram, as illustrated in **Figure 8B**. This visual representation intricately traces the evolutionary path of various keywords from 2000 to 2023. Initially, scholarly inquiries focused on unraveling the complexities of the structure and diversity within the intestinal fungal community. Over time, attention shifted towards exploring biochemical substances and reactions, illustrating the dynamic nature of scientific inquiry. Currently, research emphasis has shifted towards investigations centered around the intestinal microbiota, emerging as a focal point in this field. Our anticipation suggests that this growing interest will persist and guide research directions in the foreseeable future.

In **Figure 8C**, a bubble chart visually presents the temporal occurrences of keyword eruptions. The size of each bubble reflects the level of attention it received during specific time intervals. The emergence of keywords in 2011 can be linked to the advancement of gradient gel electrophoresis technology, enabling more cost-effective and convenient extraction of microorganisms. In 2012, a multinational, multi-laboratory consortium assessed six DNA regions and subsequently recommended the Internal Transcribed Spacer (ITS) region as the standard barcode for fungi. This recommendation established a crucial foundation for future fungal research (27). Moreover, the remarkable increase in scholarly interest in the relationship between gut microbiota and diseases like cancer and Crohn’s disease led to the peak of keyword explosion in 2021.

## 4 Discussion

### 4.1 General information

In our study, we examined 1434 articles from the Web of Science Core Collection (WoSCC) database, focusing on the intersection of fungi and gut microbiota. We employed R language, VOSviewer, and CiteSpace for data processing and visualization. The analysis revealed a significant increase in annual publication output and citation counts, particularly from 2014 onwards. China, France, and the United States emerged as prominent contributors in this field. Among the top 10 institutions by publication volume, 5 are from the USA, including the University of California System, Weill Cornell Medicine, Cornell University, Harvard University, and the University of Wisconsin-Madison, highlighting the leading position of the United States. While only one Chinese institution, the Chinese Academy of Sciences, is listed, its second-ranking in publication output underscores China’s growing attention and investment in this area. These findings may be associated with the increased funding from sources such as the National Natural Science Foundation of China, the “973” program, the “863” program (28), and initiatives supported by the Chinese Academy of Sciences.

Professor Zhang F, a prominent Chinese scholar from the Department of Food Science and Engineering at Jinan University, focuses on the relationship among food nutrition, intestinal microbiota, and human health (29). During the Covid-19 pandemic, Dr. Zhang F’s research explores changes in the gut microbiota of infected patients (30). On the Western side, Professor Iliev ID, based at Cornell University’s Mycobiota and Mucosal Immunology Laboratory, stands out for his significant study. His research reveals the complex interaction between gut fungi and the immune system through the Dectin-1 receptor, influencing susceptibility to colitis (19). This study also clarifies the association between Dectin-1 gene variations and severe ulcerative colitis, enhancing our understanding of the implications of the gut microbiome.

From a journal perspective, Frontiers in Microbiology and PLOS ONE are the leading publications, each having published over 2000 papers. Frontiers in Microbiology, renowned for its pioneering research dissemination in microbiology, maintains a distinguished reputation within the Frontiers journal series for its excellence and innovation in scientific publishing. Encompassing diverse microbiological domains such as microbial ecology, virology, bacteriology, mycology, and microbial genetics, it serves as a comprehensive platform for scholarly exchange. Meanwhile, MICROBIOME, with its prolific publication output, boasts the highest impact factor and is widely recognized as the foremost microbiology journal. Both MICROBIOME and Frontiers in Microbiology advocate for open access and scholarly collaboration, yet MICROBIOME’s focused approach distinguishes it within the field.

### 4.2 Historical evolution in the research

Through the analysis of influential papers and historical citation networks, we have gained insights into the trajectory of gut fungal community research. It began with a mouse experiment by Iliyan D. Iliev in June 2012, revealing a diverse fungal community in the mammalian gut interacting with the immune system via the Dectin-1 receptor. This study unveiled a new eukaryotic fungal community in the gut, sparking further research. Over the following five years, research on gut fungi gradually increased as scholars delved into their associations with critical life and health issues. Zelante and colleagues (17) revealed a metabolic pathway of endogenous tryptophan metabolites that resist Candida albicans colonization and shield the mucosa from inflammation. Hoffmann (22) identified associations between fungi and diet, noting positive correlations between Methanobrevibacter and Candida in high-carbohydrate diets, and negative correlations with diets rich in amino acids, proteins, and fatty acids. This supported Lewis’s milestone work (23) indicating the gut microbiota’s rapid response to dietary changes but without establishing the precise link between gut fungi and diet. The focus of gut fungal community research shifted towards inflammatory bowel disease (IBD) in 2017 due to increased recognition of the microbiome’s role in IBD, advancements in sequencing technologies, emerging evidence of fungal involvement, and heightened clinical relevance. Sokol et al. (18) conducted a large cohort bioinformatics analysis, revealing a distinct dysbiosis of fungal microbiota in IBD, characterized by an altered Basidiomycota/Ascomycota ratio, reduced Saccharomyces cerevisiae, and increased Candida albicans. This dysbiosis could be attributed to the specific intestinal milieu of Crohn’s disease, favoring fungi while hindering bacteria (8). Towards the year-end, “The Gut Mycobiome of the Human Microbiome Project Healthy Cohort” was published in the esteemed journal MICROBIOME (25), revealing ITS2 sequencing results. These indicated lower diversity in the human gut mycobiome compared to the bacterial community, primarily comprising yeasts like Saccharomyces, Malassezia, and Candida. Amid the COVID-19 pandemic, some researchers connected gut fungal research with the virus. In 2020, a study observed sustained changes in 53% (16 out of 30 patients) of fecal fungal communities during the disease process, with elevated proportions of Candida albicans, Aspergillus, and Penicillium in fecal samples throughout COVID-19 patients’ hospitalization (31). Despite the pandemic’s conclusion, research on the gut fungal community remains active. For instance, Liang, S.H et al. discovered that Candida albicans has developed hyphal-specific factors, such as candidalysin, which could potentially restrict bacterial metabolic output, thereby enhancing its competitive edge against bacterial species within the intestinal niche (32). Szóstak et al. elucidated that fungal composition and specific fungal species are linked to an elevated risk of melanoma progression and influence the response to Anti–PD-1 therapy, underscoring the pivotal involvement of gut fungi in cancer immunotherapy (33). In essence, as research advances, we anticipate a proliferation of knowledge concerning gut fungi, unraveling further complexities in this evolving field.

### 4.3 Analysis of research hotspots

Through diverse techniques including keyword WordCloud, TreeMap, trend bubble chart, fluorescence chart, and highly cited literature, we have identified the predominant research focuses on intestinal fungal communities.

#### 4.3.1 Methods for studying the human gut fungi

Historically, scientists have typically relied on culture-dependent methods to survey fungal populations within complex microbial ecosystems (34–36). These methods involve traditional microbiological techniques such as microscopy (37), biochemical assays (38), and growth on selective media (39), often using widely available media like Sabouraud dextrose and potato dextrose. However, these techniques have inherent biases and limitations, favoring rapidly proliferating species and potentially missing rare fungal diversity. Additionally, discrepancies between culture-dependent and culture-independent data further complicate analysis of microbial communities.

Advancements in molecular methodologies (40–44), including PCR, Sanger sequencing (45), and next-generation sequencing (NGS) technologies (46, 47), have liberated scientists from traditional culturing techniques in ecological surveys. These technologies offer a more cost-effective means to investigate and identify microbial communities, eliminating the dependence on culturomics (48, 49). Nevertheless, it’s crucial to acknowledge the absence of a standardized approach for culture-independent gut fungi analysis. Researchers are tackling this issue through the development of standardized protocols, the establishment of reference databases, advancements in sequencing technologies, and collaborative efforts to formulate unified guidelines.

#### 4.3.2 Fungal interactions

##### 4.3.2.1 Gut fungus and Immunity

Recent research highlights the pivotal role of Dectin-1, a crucial pattern recognition receptor (PRR), in fungal immunity. Its structure, featuring a single extracellular C-type lectin domain within a type II transmembrane architecture, enables recognition of the β-1,3 glucan motif present in fungal cell walls, eliciting a robust immune response against these pathogens. Moreover, insights from Moyes and Naglik (50) suggest Dectin-1’s involvement in colitis development via commensal fungi interaction. Recent discoveries elucidate Dectin-1’s capacity to bind β-glucans (BG), pivotal fungal cell wall components, promoting fungal cell phagocytosis and reactive oxygen species generation within macrophages. Additionally, Dectin-1 synergizes with Toll-like receptors (TLRs), notably TLR2, while independently orchestrating immune responses, emphasizing its multifaceted and indispensable role in host-fungal interactions (51). In summary, further research into the functions and interactions of these pattern recognition receptors (PRRs) is anticipated to provide valuable insights that could inform the development of immunotherapies. These therapies would aim to enhance antifungal host defenses and address the rising mortality rates associated with invasive fungal infections.

##### 4.3.2.2 Gut fungus and gut-brain axis

While much attention has been devoted to studying the gut-brain axis with a focus on intestinal bacteria, the potential impact of the gut fungal community may have been overlooked. Recent clinical and experimental research indicates that fungi could indeed play a crucial role in brain-gut communication, utilizing neuro-immuno-endocrine mediators, akin to the microbiome-gut-brain axis (11). Tao Ye’s study exemplifies mycobiome-gut-brain communication, showcasing how S. boulardii modulates the TLRs pathway and inhibits neuroinflammation via the gut-brain axis (52). Research has found that probiotic SB treatment significantly alleviates cognitive deficits, Aβ aggregation, synaptic dysfunction, neuroinflammation, intestinal barrier impairment, and fungal microbiome abnormalities in APP/PS1 mice. The potential mechanism by which probiotic SB modulates AD-related neuroinflammation may be associated with the TLRs pathway. This study lays a foundation for further investigation into the involvement and manipulation of intestinal fungi within the gut-brain axis.

##### 4.3.2.3 Fungal-bacteria Interactions

Research exploring the interaction between fungi and bacteria often involves inducing gut dysbiosis, followed by assessing outcomes via antifungal or antibacterial treatments (53). The sensitivity of Candida species to anaerobic-specific or broad-spectrum antibiotics has garnered attention in these studies (54). Jiang et al. demonstrated that commensal fungi like Candida albicans or Staphylococcus aureus can effectively substitute for intestinal bacteria during antibiotic-induced bacterial dysbiosis (55). Conversely, Mason et al. found that Candida albicans colonization during antibiotic recovery might increase lactobacilli prevention and enterococcal colonization (56). The presence of specific bacteria reducing fungal colonization opportunities is notable. Tampakakis et al. showed that Salmonella enterica serovar Typhimurium reduces both viability and colonization of Candida albicans in planktonic *in vitro* cultures (57). Additionally, certain fatty acid metabolites from bacterial communities appear to modulate Candida albicans germination. Recent studies, like García et al., suggest that gut microbial metabolites inhibit the invasion of human intestinal cells and the filamentous growth of Candida albicans via the rapamycin (TOR) signaling pathway (58).

In addition, current research underscores complex interactions in biofilm habitats, known as “mixed-species biofilms,” between fungal and bacterial cells. Fungi, like Candida species, can boost invasiveness via hyphae induction and extracellular enzyme production, including aspartic proteases. Concurrently, bacteria may gain antibiotic resistance advantages in this shared habitat. Consequently, characterized fungal-bacterial interactions can be fundamentally synergistic, antagonistic, commensal, or mutualistic (59–61).

#### 4.3.3 Gut mycobiome and disease susceptibility

The human mycobiome plays a pivotal role in gastrointestinal-related ailments such as IBD (62–64), fungal infections (15), colorectal adenomas (65), pouchitis post ileal pouch-anal anastomosis (66), and diarrhea (67), significantly impacting host health status (19, 68). For instance, fungal colon infections arise when impaired immune responses, including deficiencies in IL-22, IL-23, and Th1/Th17 cell functions, fail to effectively control fungal growth and dissemination (69). **Table 6** outlines the substantial contribution of fungi to human diseases.

**Table 6:** Substantial implication of fungi in human disease progression.

Disease	Disease subtypes	Significant findings that involved fungi
Inflammatory bowel disease	Crohn’s disease (CD)	(i) Fungal dysbiosis is closely related to CD in most of the conducted studies
		(ii) Interkingdom interaction between fungal and bacteria was observed
Inflammatory bowel syndrome (IBS)	—	(i) Fungal dybiosis, predominant by *Saccharomyces cerevisiae* and *Candida albicans* in IBS patients
Cancers	Colorectal cancer	(i) Fungal dysbiosis is observed in most of the reported studies
Infectious diseases	Hepatitis B	(i) High levels of *Aspergillus*, *Candida*, *Galactomyces*, *Saccharomyces*, and *Chaetomium* were identified
		(ii) Richness and diversity of fungal species is associated with chronic HBV infection
	HIV	(i) *C. parvum*, *C. difficile*, and *C. albicans* are significantly present in HIV-seropositive patients
		(ii) *C. albicans*, *C. krusei*, and *C. tropicalis* were associated with diarrhea in HIV patients
		(iii) Fungal dysbiosis and high prevalence of *Candid*a species were observed in HIV patients
		(iv) Prevalence of *Candida* in HIV patients without antiretroviral treatment was higher than HIV patients with active antiretroviral treatment
Noncommunicable diseases	Obesity	(i) *Candida*, *Nakaseomyces*, and *Penicillium* genera were commonly identified in obese subjects
		(ii) *Mucor racemosus* and *M. fuscus* were identified in nonobese patients.
		(iii) Specific fungal composition could be potentially used to distinguish between obese and nonobese patients
	Diabetes	(i) *C. albicans* is more prevalence in type 1 diabetes
		(ii) *C. albicans* is more prevalence in type 1 and type 2 diabetes
		(iii) No difference is found between *C. albicans* colonization in type 1 and type 2 diabetes.
		(iv) Isolated fungal species from type 1 diabetes patient is more resistant towards antifungal treatment
Atherosclerosis	—	(i) Phylum *Zygomycota*, which consists of family *Mucoraceae* and genus *Mucor*, was negatively correlated with the risk of cardiovascular disease development through carotid intima-media thickness (cIMT) method
Alcoholic liver disease	—	(i) Decreased in fungal diversity along with *Candida* overgrowth in alcohol-dependent patients
Central nervous system diseases	Rett syndrome	(i) High abundance of *Candida* genus were detected
	Autism Spectrum disorder	(i) *Candida*, *Malassezia*, *Aspergillus*, and *Penicilliun* genera were identified
	Schizophrenia	(i) Increased levels of *S. cerevisiae* and *C. albicans* species
		(ii) Close association of gastrointestinal tract disturbance with elevation of *C. albicans* species and lower cognitive score

#### 4.3.4 Human microbiome project healthy cohort

In a study led by Nash et al., the gut fungal community was assessed via analysis of the ITS2 region and the 18S rRNA gene within the Human Microbiome Project (25). Results indicated lower diversity in fecal fungi compared to bacteria, with yeasts being prevalent, especially among the top 15 genera. Notably, Malassezia and Candida were prominent within the fungal community. Various OTUs, including Saccharomyces cerevisiae and Candida albicans, were widely present. Unlike bacteria, fungal diversity showed significant variability, albeit with some species consistently observed across samples from the same subjects.

### 4.4 Limitations

This study has several limitations. Firstly, we adhered to bibliometric analysis guidelines by utilizing only the Web of Science (WoS) database, a major biomedical database (70). However, this approach may have resulted in the exclusion of relevant publications not indexed in WoS. Secondly, our analysis is limited to English-language publications, which may overlook significant non-English research. Thirdly, the limitations of search keywords could introduce bias, potentially leading us to miss emerging areas of focus; in the future, we might incorporate artificial intelligence to address keyword drift issues. Additionally, the ambiguous nomenclature of gut mycobiome communities means that some articles might instead address gut bacterial communities. Lastly, the selected search terms may not capture all relevant documents.

## 5 Conclusions

Utilizing the R language’s Bibiometrix package, VOSviewer, and CiteSpace software, we conducted a bibliometric analysis of gut microbiome fungal research. Visual results depict a steady increase in research volume since 2000, with a notable surge post-2014, indicating a growing interest in gut fungi. China leads in publication volume, while the United States produces high-quality articles. Despite being a research hotspot, the molecular mechanisms linking gut fungal communities with bacterial communities, the immune system, the gut-brain axis, and diseases remain unclear and require further elucidation. Remarkably, the recent advent of third-generation sequencing technologies and artificial intelligence is poised to substantially advance research on gut fungi. We anticipate that future research will shed light on the overlooked significance of the gut fungal community within the gut microbiome.

## Data Availability

The original contributions presented in the study are included in the article/supplementary material. Further inquiries can be directed to the corresponding author.
